# Machine learning-based disulfidptosis-related lncRNA signature predicts prognosis, immune infiltration and drug sensitivity in hepatocellular carcinoma

**DOI:** 10.1038/s41598-024-54115-8

**Published:** 2024-02-22

**Authors:** Lei Pu, Yan Sun, Cheng Pu, Xiaoyan Zhang, Dong Wang, Xingning Liu, Pin Guo, Bing Wang, Liang Xue, Peng Sun

**Affiliations:** 1 The Key Laboratory of Adolescent Health Assessment and Exercise Intervention of the Ministry of Education, East China Normal University, 500 Dongchuan Road, Shanghai, 200241 People’s Republic of China; 2 Department of Veterinary Medicine, Shandong Vocational Animal Science and Veterinary College, Weifang, 261071 Shandong People’s Republic of China; 3 School of Martial Arts, Shanghai University of Sport, Shanghai, 200438 People’s Republic of China; 4 Department of Oncological Surgery, Minhang Branch of Shanghai Cancer Center, Fudan University, Shanghai, 200240 People’s Republic of China; 5 Zhejiang Institute of Sports Science, Hangzhou, 310004 Zhejiang People’s Republic of China; 6 Jiangsu Vocational Institute of Architectural Technology, Xuzhou, 221116 Jiangsu People’s Republic of China

**Keywords:** LncRNA, Hepatocellular carcinoma cells, Disulfidptosis, Immune microenvironment, Prognostic signature, Cancer, Computational biology and bioinformatics

## Abstract

Disulfidptosis a new cell death mode, which can cause the death of Hepatocellular Carcinoma (HCC) cells. However, the significance of disulfidptosis-related Long non-coding RNAs (DRLs) in the prognosis and immunotherapy of HCC remains unclear. Based on The Cancer Genome Atlas (TCGA) database, we used Least Absolute Shrinkage and Selection Operator (LASSO) and Cox regression model to construct DRL Prognostic Signature (DRLPS)-based risk scores and performed Gene Expression Omnibus outside validation. Survival analysis was performed and a nomogram was constructed. Moreover, we performed functional enrichment annotation, immune infiltration and drug sensitivity analyses. Five DRLs (AL590705.3, AC072054.1, AC069307.1, AC107959.3 and ZNF232-AS1) were identified to construct prognostic signature. DRLPS-based risk scores exhibited better predictive efficacy of survival than conventional clinical features. The nomogram showed high congruence between the predicted survival and observed survival. Gene set were mainly enriched in cell proliferation, differentiation and growth function related pathways. Immune cell infiltration in the low-risk group was significantly higher than that in the high-risk group. Additionally, the high-risk group exhibited higher sensitivity to Afatinib, Fulvestrant, Gefitinib, Osimertinib, Sapitinib, and Taselisib. In conclusion, our study highlighted the potential utility of the constructed DRLPS in the prognosis prediction of HCC patients, which demonstrated promising clinical application value.

## Introduction

According to Global Cancer Statistics^1^, in 2020, there were 905,677 new cases of liver cancer and 830,180 new deaths, accounting for 4.7% of all cancer cases and 8.3% of all cancer-related deaths respectively, and ranking as the third cause of cancer-related mortality. Live cancer mainly includes hepatocellular carcinoma (HCC), intrahepatic cholangiocarcinoma, and mixed liver cancer, among which HCC is the most commonly diagnosed primary cancer of the liver, comprising 75–85% of cases of all liver cancer cases. Presently, although hepatic resection, liver transplantation, radiotherapy, chemotherapy and targeted therapy have improved the clinical prognosis for some HCC patients, the prognosis of HCC remains poor, with a post-operative recurrence rate of up to 70%^2^. Therefore, exploring novel prognostic models and potential therapeutic targets is of great clinical significance.

In March 2023, Liu et al.^3^ first reported in the journal Nat Cell Bio a new form of programmed cell death, which may serve as a new targeted therapeutic approach. The study found that cell death still occurred after the knock-down of genes that regulated apoptosis and iron death, but was inhibited by the use of the thiol-based reducing reagent Tris (2-Carboxyethyl) Phosphine (TCEP). Further analysis revealed that SLC7A11, which was highly expressed in cancer cells, mediated the uptake of cystine and consequently the synthesis of glutathione to counteract the oxidative effect caused by high expression. However, under glucose starvation, the supply of nicotinamide adenine dinucleotide phosphate (NADPH) generated through the pentose phosphate pathway was insufficient, and would lead to the inhibition of the cysteine reduction to cysteine pathway and the increase of intermolecular disulphide bonds of actin, which induced disulphide stress and activated the Rac-WAVE regulatory complex (WRC)-actin-related protein (Arp2/3) signaling axis, triggering cytoskeletal disorders and cell death. In this process, excessive cystine intake combined with glucose starvation may induce cell death. Due to such stress characteristics of disulfide compounds, this form of cell death was termed disulfidptosis. It reported that GYS1, NDUFS1, OXSM, LRPPRC, NDUFA11, NUBPL, NCKAP1, RPN1, SLC3A2, and SLC7A11 were closely associated with disulfidptosis.

Long non-coding RNAs (lncRNAs) refer to a type of non-coding RNAs more than 200 nucleotides in length, which are distributed in the nucleus and cytoplasm and can regulate the biological behavior of cancer cells by binding to DNA, RNA and protein, such as apoptosis, proliferation and metastasis. Numerous studies have reported that lncRNAs are closely associated with the occurrence and development of hepatocellular carcinoma. Zhou et al.^4^ found that lncRNA PART1 promoted HCC proliferation, migration and invasion by regulating miRNA-149-5p/MAP2K1 Axis. Wei et al.^5^ found that lncRNA PAARH inhibited HCC apoptosis and promoted HCC migration, invasion and tumor growth by upregulating HOTTIP and activating HIF-1α/VEGF pathway. In addition^6^, lncRNAs are found to be closely associated with programmed cell death. A study found that the prognostic model based on ferroptosis-related lncRNAs (POLH-AS139384, MKLN1-AS, AC090772.3, etc.) could accurately predict the prognosis of HCC patients. The above studies have demonstrated the potential critical role of lncRNAs in the treatment and prediction of HCC, but the role of DRL in HCC remains to be elucidated.

At present, in studies on the modes of cell death of HCC, Ferroptosis-Related lncRNA^7^, Cuproptosis-Related lncRNA^8^, pyroptosis-related lncRNA^9^, necroptosis-related lncRNAs^10^ all provide valuable prognostic biomarkers and therapeutic targets for HCC. However, DRLs remain unclear, so comprehensive evaluation of DRLs and construction of prognostic markers are essential for accurate treatment of clinical diagnosis, such as monitoring of prognostic markers, early detection of disease changes, and adoption of appropriate therapeutic measures to improve the survival and life quality of patients. Therefore, in this study, we used R to perform a comprehensive analysis of TCGA-LIHC-RNA-seq data and clinical data, identifying prognostic-related DRLs in HCC and constructing a prognostic risk-scoring model. In addition, a nomogram was constructed based on DRLPS risk scores to assess OS in HCC patients. Finally, we evaluated immune infiltration and drug sensitivity in high- and low-risk groups. These results were expected to provide references for the prognosis, immunotherapy and its mechanism in HCC.

## Results

### Identification of DRLs in TCGA-LIHC

**Figure 1:**
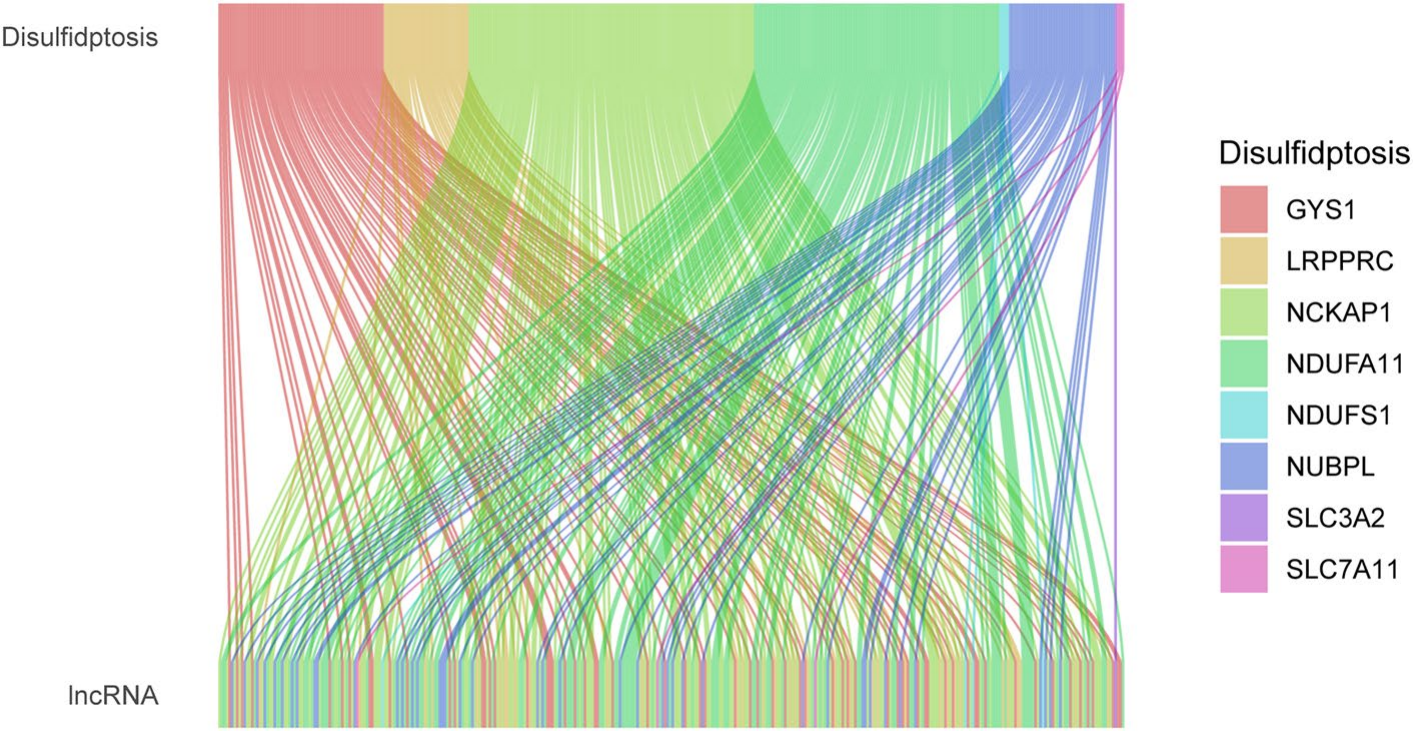
The Sankey diagram presents detail connections between DRLs and DRGs.

Based on TCGA-LIHC-RNA-seq data, 50 normal tissue samples, 374 HCC samples, and 16,876 lncRNA expression data (Supplementary Table S1) were extracted. Currently, only Liu et al.^3^ reported that GYS1, NDUFS1, OXSM, LRPPRC, NDUFA11, NUBPL, NCKAP1, RPN1, SLC3A2, and SLC7A11 were closely associated with disulfidptosis. Excluding normal tissue samples, 362 DRLs (Supplementary Table S2) were obtained by co-expression analysis (Fig. 1) and were intersected with survival data (survival time, survival status) of TCGA-LIHC to obtain 317 lncRNAs and 370 HCC samples (Supplementary Table S3).

### Construction of DRLPS in the training set

**Figure 2:**
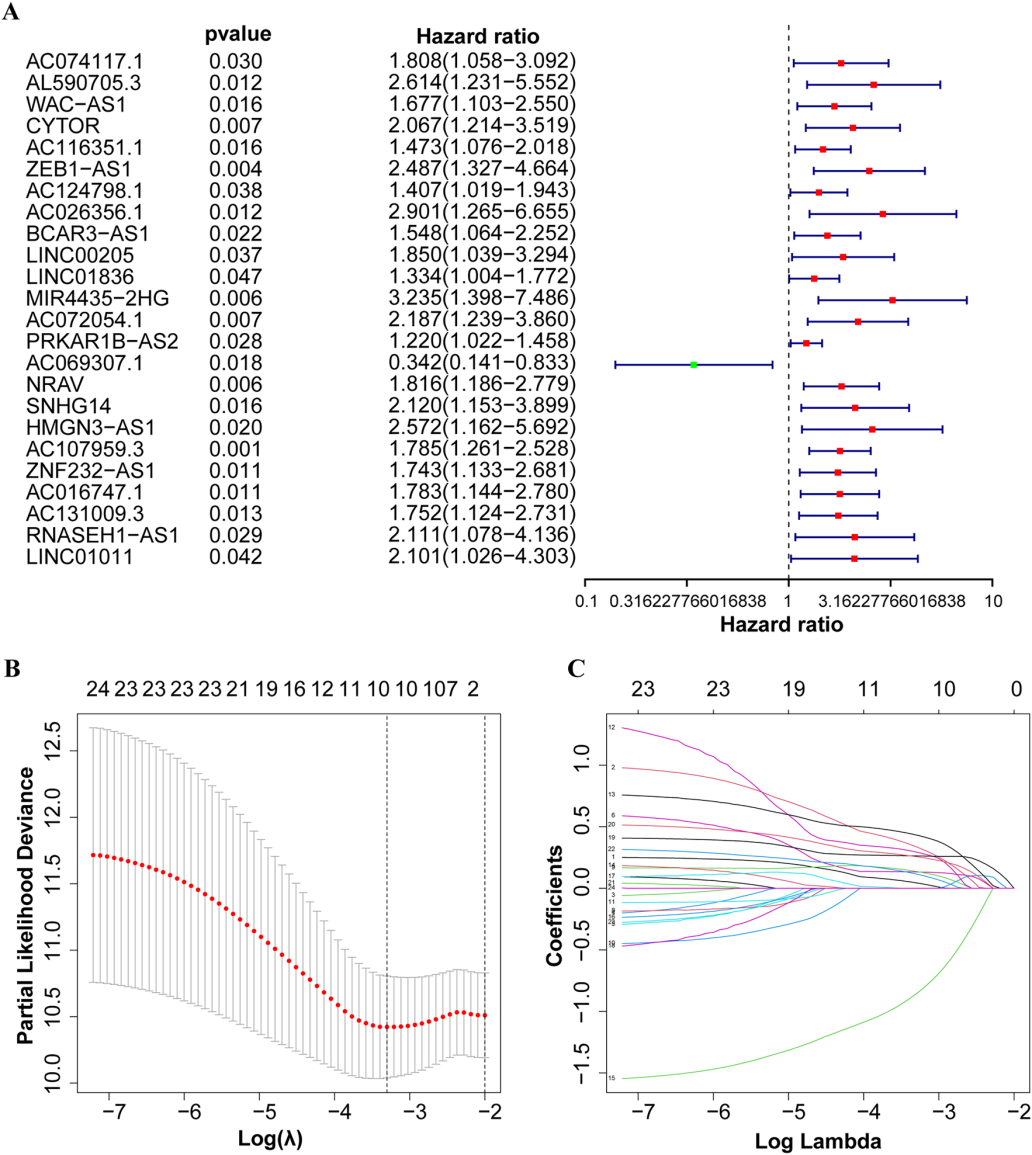
Identification of prognostic-related DRLs and construction of DRLPS based on the training set. (**A**) Univariate COX regression analysis to identify prognosis-related lncRNAs. (**B, C**) Lasso regression to identify 10 candidate lncRNAs.

The 370 samples were grouped into training and testing sets in a 1:1 ratio using the random function in R software, and were tested for intra-group comparability. The clinicopathological features of the two sets of samples were shown in Supplementary Table S7, with all *p*-values > 0.05, indicating statistical comparability. 24 prognosis-related lncRNAs were identified by univariate Cox regression (*p* < 0.05) (Fig. 2A). Among them, AC069307.1 (HR: 0.342, 95%-CI: 0.141–0.833) was protective lncRNA; the rest (AC074117.1, AL590705.3, WAC-AS1, CYTOR, AC116351.1, ZEB1-AS1, AC124798.1, AC026356.1, BCAR3-AS1, LINC00205, LINC01836, MIR4435-2HG, AC072054.1, PRKAR1B- AS2, NRAV, SNHG14, HMGN3-AS1, AC107959.3, ZNF232-AS1, AC016747.1, AC131009.3, RNASEH1-AS1, LINC01011) were all risk lncRNAs. Lasso regression with ten-fold cross-validation was conducted, identifying 10 candidate lncRNAs (AC074117.1, AL590705.3, ZEB1-AS1, BCAR3-AS1, MIR4435-2HG, AC072054.1, AC069307.1, AC107959.3, ZNF232-AS1, AC131009.3) (Fig. 2B, C). The candidate lncRNAs were further screened by multivariate Cox regression analysis for lncRNAs with the greatest prognostic value, and 5 DRLs were ultimately selected to construct the optimal prognostic model. DRLPS-based risk Score = AL590705.3Expi* (0.619) + AC072054.1Expi* (0.644) + AC069307.1Expi* (− 1.302) + AC107959.3Expi* (0.493) + ZNF232-AS1Expi* (0.498).

### Validation of the prognostic value of DRLPS

Based on the median value (0.903) of risk score in the training set, the samples were divided into high-risk and low-risk groups. The results of PCA indicated that lncRNAs in the model was able to distinguish high-risk groups from low-risk groups (Supplementary information Fig. S1D). The risk score plot (Supplementary information Fig. S1G), the scatter plot of OS status (Supplementary information Fig. S1H), and heat map of the expression of 5 DRLs in the high- and low-risk groups (Supplementary information Fig. S1I), showed that the mortality rate gradually increased as the risk scores increased. K–M survival analysis (Supplementary information Fig. S1A) showed that OS in the low-risk group was higher than in the high-risk groups. Area under the curve (AUC) is an indicator for assessing model performance, with a range of 0.5–1. AUC < 0.5 indicates that the model has no application value, while 0.5 < AUC < 1 indicates that the model has certain clinical predictive value, and higher value indicates better predictive ability. Our results showed that AUC were 0.714, 0.805, and 0.822 for 1-, 3-, and 5-year survival, respectively (Supplementary information Fig. S1B), suggesting that the model had high prognostic value in the prognosis of HCC patients.

Compared with traditional clinical features (Supplementary information Fig. S1C), it was found that the risk model (AUC = 0.805) showed superior predictive efficacy than other clinical features, such as Age (AUC = 0.506), Gender (AUC = 0.435), Grade (AUC = 0.566), and Stage (AUC = 0.655). Univariate Cox regression revealed that the risk model was significantly associated with OS (P < 0.001; HR = 1.411, 95%-CI: 1.224–1.627) (Supplementary information Fig. S4E), and multivariate Cox regression analysis confirmed it as an independent prognostic factor in HCC patients (P < 0.001; HR = 1.343,95%-CI: 1.145–1.577) (Supplementary information Fig. S1F).

### Validation of DRLPS based on the testing set and external dataset

The testing set was applied to validate the robustness of the risk model constructed in the training set. Based on the median value in the training set, the testing set was divided into high- and low-risk groups. PCA analysis (Supplementary information Fig. S2D) demonstrated reliable clustering ability of risk score. K–M survival curves (P = 0.002) (Supplementary information Fig. S2A), Time-ROC curves (1-years AUC = 0.689; 3-years AUC = 0.635; 5-years AUC = 0.643) (Supplementary information Fig. S2B), and the risk score (AUC = 0.635) (Supplementary information Fig. S2C) had better predictive efficacy than Age (AUC = 0.502), Gender (AUC = 0.452), and Grade (AUC = 0.498). The risk score plot, scatter plot of OS status and heat map of the expression of 5 DRLs (Supplementary information Fig. S2G–I), together with univariate (P = 0.003; HR = 1.277, 95%-CI: 1.087–1.499) (Supplementary information Fig. S2E) and multivariate Cox regression analyses (P = 0.014; HR = 1.264, 95%-CI: 1.048–1.523) (Supplementary information Fig. S2F) all suggested that the results in the training set were robust. Then, to further validation the performance of DRLPS, we used 115 patient samples from an external dataset. We found that all the validation results were consistent with the Train set results, specifically, based on the median value in the GSE76427 dataset, PCA could clearly differentiate between high and low risk groups (Supplementary Fig. S3D), indicating good clustering reliability. K–M survival curves (P = 0.005) indicated that the survival of the low-risk group was significantly better than that of the high-risk group (Supplementary Fig. S3A). Both multivariate (P < 0.001, HR = 1.677, 95% CI: 1.244–2.261) (Supplementary Fig. S3F) and univariate (P < 0.001, HR = 1.715, 95% CI: 1.258–2.338) (Supplementary Fig. S3E) analyses showed that the predictive ability of the risk model could be independent of other clinical features. Time-ROC curves (1-year AUC = 0.699; 3-year AUC = 0.692; 5-year AUC = 0.798) (Supplementary Fig . S3B) and the risk score (AUC = 0.692) (Supplementary Fig. S3C) were consistent with the results in the training set. Risk curves (Supplementary Fig. S3G), scatter plots (Supplementary Fig. S3H), and DRL expression heat maps (Supplementary Fig. S3I) suggested mortality increased with risk.

### Establishment of a predictive nomogram for HCC patients

**Figure 3:**
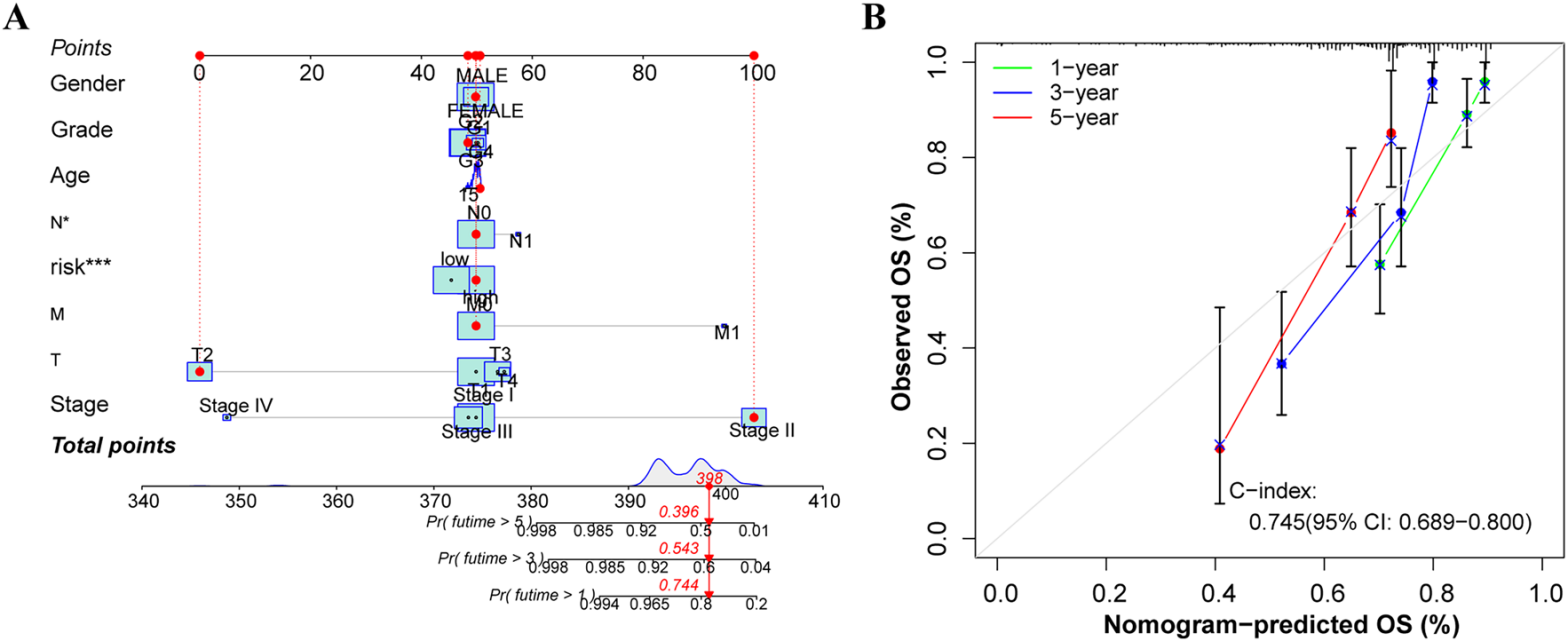
Construction of a DRLPS-based nomogram in all HCC patients for predicting OS. (**A**) Nomogram predicting 1-, 3- and 5-year OS. (**B**) Calibration curve for the nomogram.

Considering the universality of the risk model, a nomogram was developed using DRLPS-based risk score of 370 LIHC samples in conjunction with clinical features (Gerder, Grade, Age, TMN Stage) (Fig. 3A). The calculated consistency index (C-index) was 0.745 (95%-CI: 0.689–0.8), indicating high accuracy of the nomogram in predicting OS of HCC patients (Fig. 3B).

### Go and KEGG enrichment analysis

**Figure 4:**
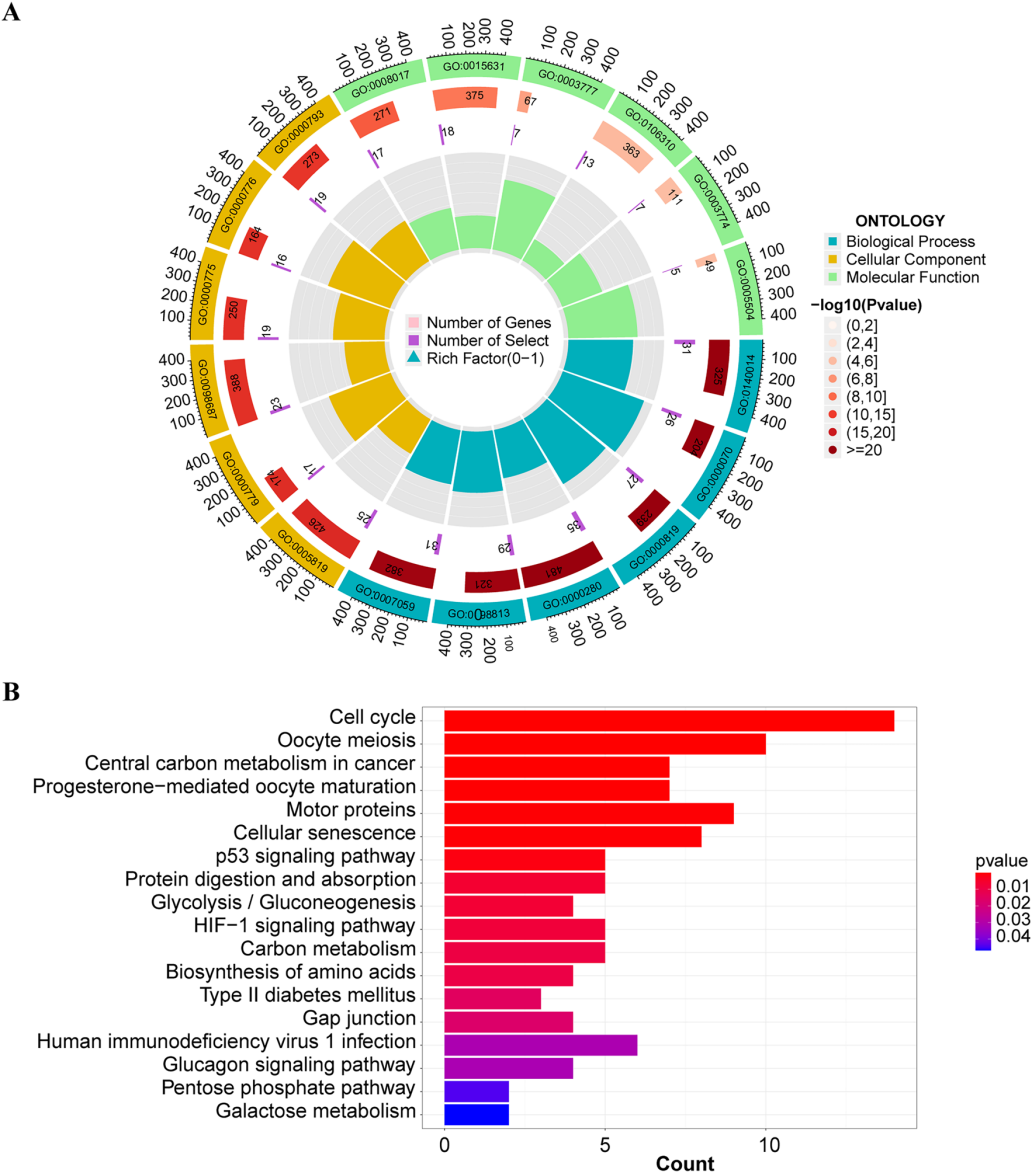
Biological functional and pathway enrichment analysis of high- and low-risk groups based on DRLPS. (**A**) GO analysis of DEGs in high- and low-risk groups. (**B**) KEGG analysis of DEGs in high- and low-risk groups.

To further investigate the potential biological functions between high-risk and low-risk groups, we performed a functional enrichment analysis of GO and KEGG pathways for mRNAs that were co-expressed with the OS-related lncRNAs in the 370 LIHC samples. There are 211 DEGs(|log2FC|> 1 and *P* < 0.05) between low and high-risk groups (Supplementary Table S4). In the GO functional enrichment analysis (Fig. 4A), the top 6 enriched biological processes (BP) were mitotic nuclear division (GO:0140014), mitotic sister chromatid segregation (GO:0000070), sister chromatid segregation (GO:0000819), nuclear division (GO:0000280), nuclear chromosome segregation (GO:0098813), and chromosome segregation (GO:0007059). The top 6 enriched Molecular Function (MF) were microtubule binding (GO:0008017), tubulin binding (GO:0015631), microtubule motor activity (GO:0003777), protein serine kinase activity (GO:0106310), cytoskeletal motor activity (GO:0003774), and fatty acid binding(GO:0005504). The top 6 enriched Cellular Component (CC) were spindleGO:0005819condensed chromosome, centromeric region (GO:0000779), chromosomal region (GO:0098687), chromosome, centromeric region (GO:0000775), kinetochore (GO:0000776), and condensed chromosome (GO:0000793) (Supplementary Table S5).

In the KEGG pathway enrichment analysis (Fig. 4B), the top 6 enriched pathways were Cell cycle, Oocyte meiosis, Central carbon metabolism in cancer, Progesterone-mediated oocyte maturation, Motor proteins, and Cellular senescence (Supplementary Table S6).

### Immune infiltration and drug sensitivity analysis

**Figure 5:**
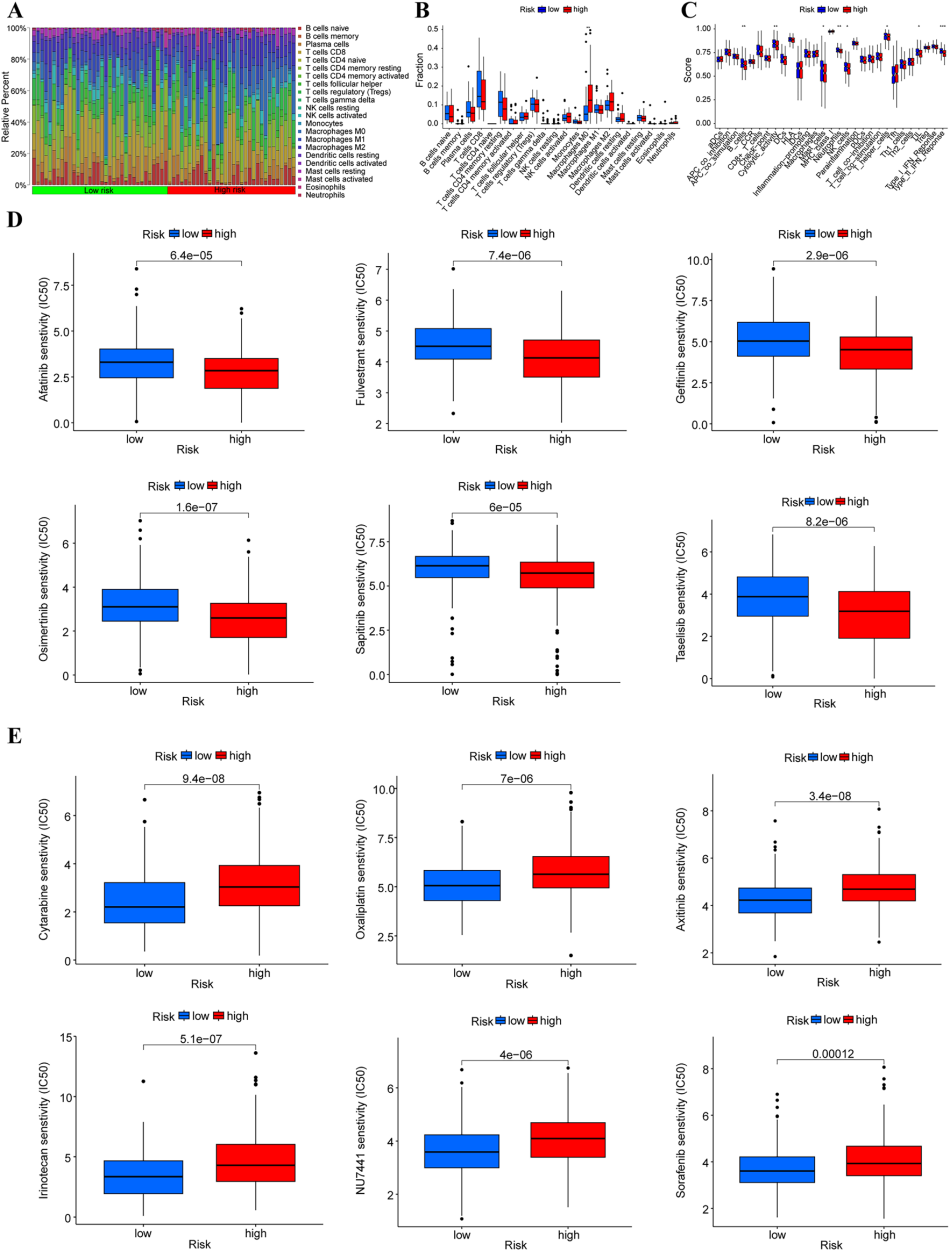
DRLPS-based analysis of immune infiltration and drug sensitivity. (**A**) Heat map of immune infiltration abundance in high- and low-risk groups. (**B**) Box plot of the differences in immune cell abundance between high- and low-risk groups. (**C**) Box plot of differences in immune function between high- and low-risk groups. (**D**) Drugs with more sensitivity in the high-risk group. (**E**) Drugs with more sensitivity in the low-risk group.

CIBERSORT algorithm was used to evaluate the abundance of 22 different immune cell types between high and low risk group in 370 LIHC samples (Fig. 5A). It was found that the infiltration abundance of M0 Macrophages in the high-risk group was significantly higher than that in the low-risk group (Fig. 5B). Through ssGESA analysis, we quantified the enrichment scores of immune-related functions in the two risk groups. The results showed that B-cells, cytolytic activity, mast cells, neutrophils, NK cells, T helper cells, Tumor-Infiltrating T Lymphocytes (TIL), Type II interferon responses (Type II IFN Response, IFN-γ) in the low-risk group were significantly higher than those in the high-risk group (Fig. 5C), which suggested an impaired anti-tumor immune function in the high-risk group. Finally, based on GDSC database, drug sensitivity analyses were performed on both high- and low-risk groups. The 50% inhibition concentration (IC50) is used to reflect anti-tumor drug sensitivity. Drug sensitivity increases as IC50 value decreases, with lower IC50 values indicating higher sensitivity and better therapeutic efficacy. It was found that the high-risk group exhibited higher sensitivity to Afatinib, Fulvestrant, Gefitinib, Osimertinib, Sapitinib, and Taselisib (Fig. 5D), while the low-risk group exhibited higher sensitivity to Cytarabine, Oxaliplatin, Axitinib, Irinotecan, NU7441, Sorafenib (Fig. 5E).

## Discussion

HCC is the most representative primary cancer of liver with high morbidity and mortality^11^. Currently, surgical therapy and chemotherapy remain the major options for HCC patients. However, the insidious pathogenesis, complex etiology, and strong invasiveness lead to poor clinical prognosis for most patients. Recently reported disulfidptosis may serve as a targeted therapeutic approach for tumors. The high expression of SLC7A11 in cancer cells results in excessive uptake of cystine into the cell, the reduction process of which consumes large amounts of NADPH (glucose metabolite) and induces cell-specific death by inhibiting glucose transporter. lncRNAs can play a regulatory role in programmed cell death through gene transcription and post-transcriptional modification, and act as potential therapeutic targets and biomarkers for the prediction and diagnosis of cancer patients, e.g., ferroptosis-related lncRNAs signature^12^, and cuproptosis-related lncRNAs signature^13^ in the prognosis of HCC. However, it is not yet clear the value and significance of DRLs in the prediction of prognosis of HCC patients. Therefore, this study constructed a DRLPS based on machine learning and explored the biological pathways, immune infiltration and chemotherapy drugs in HCC patients with the aim of improving the OS.

To the best of our knowledge, this study is the first to report the value and significance of DRLPS in the prognosis, immune infiltration and drug sensitivity in HCC patients. Upon analysis, we identified a total of 362 DRLs, among which 24 DRLs were found to have potential prognostic value by univariate Cox analysis. Among them, AC069307.1 was an important protective factor, but has not been reported in any study. It was worth noting that our co-expression analysis was very similar to the weighted gene co-expression network analysis by Zhang et al.^14^. Overall, Zhang et al., used a global approach to construct co-expression network of oral squamous cell carcinoma (OSCC) and predicted the pathogenesis of OSCC and gene clusters of tumorigenesis, which was also a novel perspective and provided multiple core targets. However, the difference is that we focused only on a risk model consisting of specific lncRNAs. Despite the different types of cancers studied by us and Zhang et al., it has been shown that both techniques may be used for a wide range of other cancers, such as breast cancer, melanoma, and others. Using Lasso regression with ten-fold cross-validation to avoid overfitting and multivariate Cox regression analysis, 5 DRLs were finally selected to construct prognostic signature, including AL590705.3, AC072054.1, AC069307.1, AC107959.3, and ZNF232-AS1. Studies reported^9,15^ that AL590705.3 was closely associated with DNA damage repair and pyroptosis, and could be used as a biomarker for the prediction and diagnosis of gastric cancer and HCC. Notably, AC072054.1 and ZNF232-AS1 have not been reported in previous studies and may be new markers. Huang et al.^16^ reported that AC107959.3 was one of the important prognostic targets for predicting hepatitis virus-positive HCC patients. Based on the median value of risk score, HCC patients were divided into high-risk and low-risk groups. PCA was used to visualize distinction between the high- and low-risk groups. K–M analysis showed that OS in the high-risk group was significantly lower than that in the low-risk group. Multivariate Cox regression showed that the constructed risk score model was a prognostic factor independent of other clinical features (Age, Gender, Grade, and Stage). The area under the ROC curve indicated good predictive efficacy of the DRLPS-based risk model, which was further validated by the testing set and outside validation set. Finally, the constructed nomogram showed a high degree of congruence between predicted 1-, 3- and 5-year OS and observed OS, indicating that the model could be used for clinical diagnosis of HCC patients. Although earlier studies reported some biomarkers and prognostic analyses for HCC, which were similar to our study, however, their shortcomings were quite obvious: they did not use more methods to investigate the dimensionality reduction, accuracy, and generalization ability of the model^17^. In this study, we used multiple machine learning approaches to improve the accuracy of biomarkers as well as their generalization ability in clinical research. In addition, early studies focused on the construction of Ferroptosis, apoptosis, and Cuproptosis prognostic markers, while Disulfidptosis is a glucose-dependent mode of death, based on which the DRLPs we constructed may provide great clinical treatment windows and new treatment ideas. For example, in interference models, the DRLs regulate the disulfidptosis-related mRNA or inhibit glucose transporter proteins by competitively binding to miRNAs, thus exerting anti-tumor effects.

Subsequently, to further reveal the differential biological functions and pathways between high- and low-risk groups, we performed GO function and KEGG pathway analyses. The results demonstrated that the top 6 biological processes were all associated with mitosis. The main proliferation mode of cancer cells is mitosis, and mitotic blockade has become an important breakthrough point in cancer-related clinical research, where attempts have been made to inhibit the proliferation, migration and invasiveness cancer cells through RNA interference technology or small molecule inhibitors. This result further suggests that lncRNAs in the model may be potential biomarkers for therapy. KEGG showed that DEGs were mainly enriched in cell cycle, oocyte meiosis, central carbon metabolism in cancer, progesterone-mediated oocyte maturation, motor proteins and cellular senescence pathway. Notably, cell cycle was the most significantly enriched pathway and an important cancer pathway, regulating cell growth, proliferation and differentiation. The four phases (G1-M) are mainly regulated by the conserved cyclin-dependent kinase (CDK)-cyclin protein complex, and are subject to cell cycle checkpoints^18^. Disruption of the cell cycle may lead to cell cycle arrest, DNA damage, abnormal spindle assembly, etc., causing abnormal cell proliferation and promoting the occurrence and development of tumor. Currently, commonly used chemotherapy drugs are designed to directly damage DNA and thus slow down cancer progression, but DNA repair mechanisms and cell cycle checkpoints are the main factors of drug resistance.

At present, immunotherapy is one of the main treatments for HCC patients, but the development of immunotherapy vastly depends on the recognition of immune microenvironment of the tumor^19^. To this end, we further analyzed immune infiltration in high- and low-risk groups. It was found that the infiltration abundance of M0 Macrophages in the high-risk group was significantly higher than that in the low-risk group. Study found that M0 macrophages in tumors prevent T cells from attacking tumor cells and secrete growth factors that encourage tumor angiogenesis^20^. Study reported that high infiltration of M0 macrophages in HCC patients showed a poor overall OS^21^. This result is consistent with the study of Jiang et al.^22^, which analyzed the association of risk scores constructed by hypoxia associated genes and immune infiltration, and found that HCC patients in the low-risk group had significantly higher M0 macrophages than those in the high-risk group. Although we found differential levels of immune cell infiltration, it was not clear how immune cell function differed between the high- and low-risk groups. Our study further identified differences in B cells, cytolytic activity, mast cells, neutrophils, NK cells, T helper cells, TIL, Type II IFN Reponse. B cells are the main effector cells of humoral immunity and are able to promote T cell response by secreting immunoglobulins, which may directly lyse tumor cells to suppress tumor progression^23^. According to Garnelo et al.^24^, tumor-infiltrating B cells and T cells were observed in close contact with each other in tumor tissues, and the functional interactions between them contributed to better prognosis of HCC patients. In addition, the immune functions of cytolytic activity^25,26^, mast cells^27,28^, neutrophils^29,30^, NK cells^31,32^, T helper cells^33,34^, TIL^35,36^, and Type II IFN Reponse^37,38^ were significantly higher in the low-risk group than those in the high-risk group, suggesting that the low-risk group has better immune microenvironment and prognosis than the high-risk group. Intriguingly, the function of neutrophils in the low-risk group was significantly better than that in the high-risk group, which was similar to the immune infiltration results of Yuan et al.^39^. They constructed a prognostic model for copper metabolism-related lncRNAs in HCC and subsequently found in immune-related analyses that Neutrophils were significantly higher in the low-risk group than in the high-risk group, which they did not explain. Neutrophils normally suppress the immune system's response to cancer and their elevation is associated with a poor prognosis of cancer. Further analyses revealed that neutrophils are able to kill tumor cells by stimulating the production of Reactive Oxygen Species, which further damage DNA and proteins and cause cell death. Finally, we identified 12 FDA-approved drugs. Among them, Afatinib^40^, Fulvestrant^41^, Gefitinib^42^, Osimertinib^43^, Cytarabine^44^, Oxaliplatin^45^, Axitinib^46^, Irinotecan^47^, Sorafenib^48^, NU7441^49^, and Sapitinib^50^ have been extensively reported in numerous studies for their value in HCC, while Taselisib (GDC-0032) has not yet been reported in terms of its association with HCC. Taselisib is a novel^51^, oral P13K selective inhibitor that is currently in clinical trial and has been reported in studies of breast cancer model^52^, head and neck squamous carcinomas model^53^ and pancreatic ductal adenocarcinoma^54^. In conclusion, abovementioned findings all suggested that our constructed prognostic DRLPS could accurately predict the prognosis of HCC patients, as well as provide new therapeutic strategies.

While our study reported for the first time the prognostic and clinical therapeutic value of DRLPS in HCC, there are several limitations. Firstly, since disulfidptosis is a newly discovered form of programmed cell death, the specific mechanism of relevant lncRNAs in disulfidptosis remains to be further explored, which will be our main research orientation afterwards. In addition, since our study was mainly based on integrative bioinformatics and public databases for analyses, the lack of experimental validation is the biggest regret of this study.

## Conclusion

Our study constructed a DRLPS by disulfidptosis-related mRNA-lncRNA coexpression that had good predictive value for the prognosis of HCC patients and may help to improve the prognosis of HCC patients and provide novel approach to more personalized treatments of immunotherapy, but further experimental validation is still needed. In the future, the results of immune infiltration can be further validated by transcriptomics, immunohistochemistry and flow cytometry, and the more specific mechanism of marker lncRNA in HCC can be validated by qPCR, dual-luciferase report assay, RNA in situ Hybridization, RNA Affinity Purification, CHIP-Seq, knockdown or overexpression experiments.

## Materials and methods

### Data acquisition and processing

**Figure 6:**
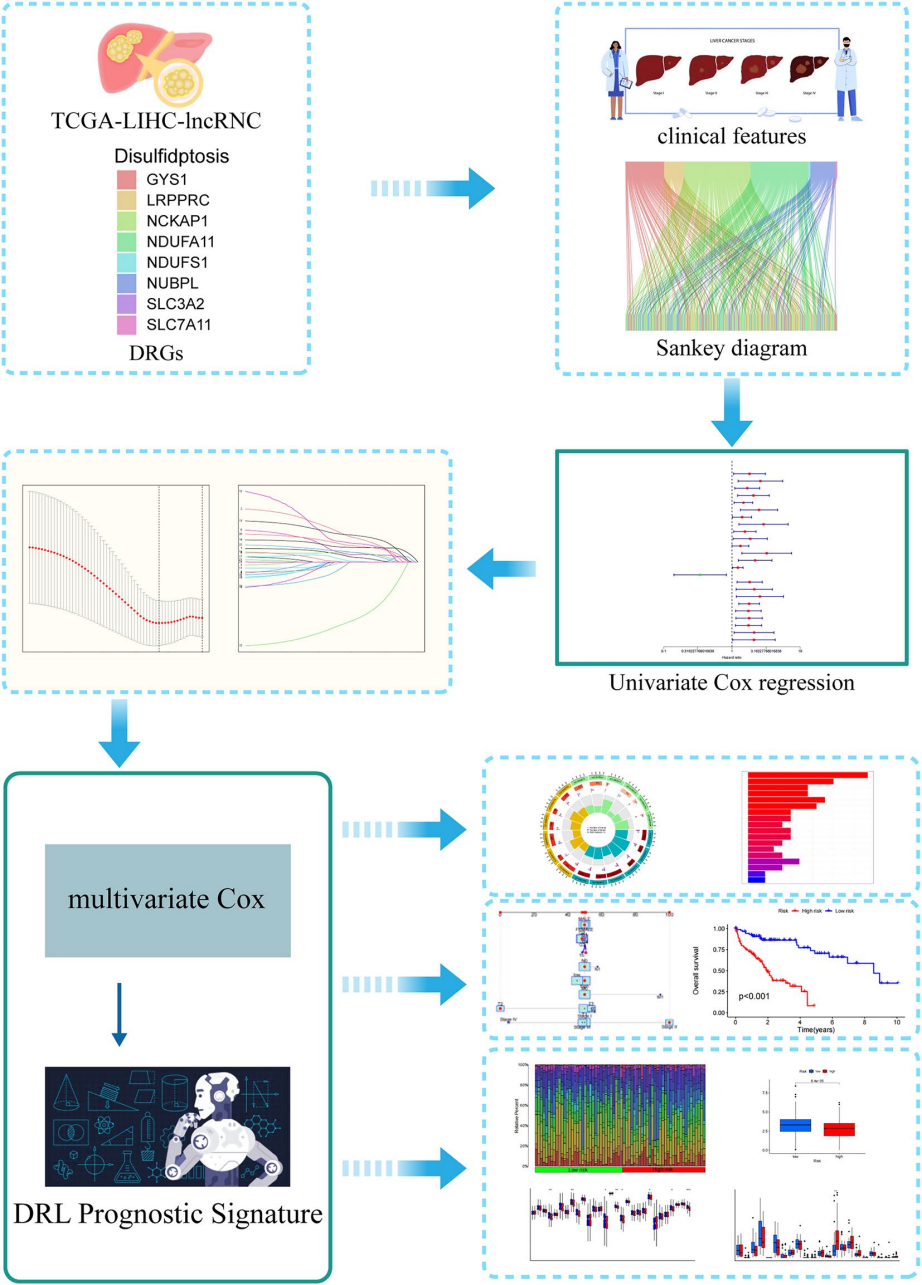
Study flowchart. In this study, 362 DRLs were identified by coexpression analysis of TCGA-LIHC-lncRNA and DRGs, and 317 DRLs and 370 HCC samples were obtained by taking the intersection with the clinical features (survival time, survival status). Univariate Cox regression was used to identify prognosis-related DRLs, which were further included in LASSO and multivariate Cox regression to construct DRLPS. Subsequently, GO, KEGG, survival analysis, immune infiltration, and drug sensitivity analyses were performed.

Figure 6 presents the research flow chart of our study design. TCGA-LIHC-RNA-seq data (which contains mRNA and lncRNA expression) and clinical data were downloaded from The Cancer Genome Atlas (TCGA) website (https://portal.gdc.cancer.gov/). Subsequently, based on the genome annotation file (GENCODE V36) provided by the R-loopBase online database (https://rloopbase.nju.edu.cn/), Strawberry Perl (Version 5.30.0.1) was used to obtain lncRNA and mRNA lists in the TCGA-LIHC-RNA-seq data and extracted expression for subsequent analysis. DRGs were obtained from previous literature^3^. DRGs expression in TCGA-LIHC-mRNA was extracted using Strawberry Perl. mRNA-lncRNA coexpression analysis of extracted DRGs and TCGA-LIHC-lncRNA expression was performed with “limma” R package to identify DRLs (|Pearson correlation coefficient (PCC)|> 0.4 and *P* < 0.001).

### Construction of DRLPS in the training set

DRLs and clinical data (survival time and survival status) were integrated using the “limma” R package. The samples (n = 1) were randomly divided into training and testing sets in a 1:1 ratio using the R software Caret package. The training set was used to train the supervised model, which adjusted the parameters according to the data in the training set and optimized the loss function to minimize the gap between the predicted and true values. The “survival” R package was used to perform univariate Cox regression analysis (P < 0.05) on the training set to identify prognostic DRLs. The core assumption of Cox regression analysis is the Proportional Hazards Assumption, i.e. Hazard Ratio (HR) maintains a constant ratio, independent of time. LASSO regression was then applied to these prognostic candidates with the “glment” R package. LASSO is a commonly used regularization in many regression analysis methods for variable selection and shrinkage in Cox’s proportional hazards model. The core principle of Lasso regression is to drive some of the regression coefficients to be strictly equal to zero by minimizing the sum of squares of the residuals under the condition that the sum of the absolute values of the regression coefficients is less than a constant, so as to reduce the complexity of the model and improve the performance and prediction accuracy. In addition, Lasso uses the sum of absolute values of the regression coefficients as a penalty function. Based on 1-SE (Standard Error) criterion, the model is trained with different penalty parameters λ, and performance is evaluated using cross-validation to find out the optimal λ. This process aims to improve the generalization ability of the model while maintaining the model fitting ability^55–57^. Therefore, the candidate DRLs were consequently identified through the optimal penalty parameter λ via the 1−SE (standard error) criterion. Finally, multivariate Cox analysis was conducted to determine the optimal DRLPS for HCC patients based on the minimum Akaike information criterion (AIC) value. AIC is a statistic used in model selection to measure the balance between the goodness of fit and the complexity of the model, with a smaller value indicating a better model. DRLPS-based risk scores were calculated using the following formula: $RiskScore = \mathop \sum \limits_{i = 1}^{N} \left( {Expi*Ci} \right)$, where *N* is the number of lncRNAs, *Expi* is the expression level of each DRL, and *Ci* is the LASSO regression coefficient.

### Evaluation and validation of DRLPS

Based on the median value of the risk score in the training set, the patients in the training and testing sets were divided into low-risk and high-risk groups. Testing set was used to examine the efficacy of the prognostic model. Testing set is obtained by differentiating the patient samples of the entire TGCA-LIHC cohort by 5:5 when the machine learning is used. It is mainly used to measure the performance of the trained model, without changing its parameters and effects, and verify whether the model was over-fitting or under-fitting, and its generalization ability when actually used. Subsequently, to further evaluate the performance of DRLPS, we used the GSE76427 dataset downloaded from the GEO database as an outside validation set. GSE76427 contained 115 HCC patient samples obtained from the GEO database, and the clinical information in it included fustat, fustime, age, gender, and stage to support our validation of DRLPS. K–M survival curve analysis, risk curves, univariate and multivariate independent prognostic analysis, ROC curve, and Principal components analysis (PCA) were performed using the “limma”, “survival”, “survivor”, “survminer”, “survivalROC”, and “timeROC” R packages to assess the prognostic model. The testing set was applied to validate this model.

### GO and KEGG enrichment analysis

ID annotation, GO and KEGG enrichment analyses were performed by “org.Hs.eg.db” and “clusterProfiler” R package with the screening criteria of |log_2_(fold change) |> 1 and FDR < 0.05.

### Immune infiltration analysis

The CIBERSORT (http://CIBERSORT.stanford.edu/) algorithm was used to evaluate the infiltration abundance of 22 immune cells between the high-risk and low-risk groups^58^. Obtaining immune.gmt from http://www.gsea-msigdb.org/gsea/index.jsp, ssGSEA was used to assess immune function in the high-risk and low-risk groups.

### Drug sensitivity analysis

Expression data and pan-cancer drug sensitivity data were obtained from the Genomics of Drug Sensitivity in Cancer database (www.cancerRxgene.org), and drug sensitivity analyses were performed on the high and low risk groups using the “oncoPredict” R package.

### Statistical analysis

R software (Version 4.2.3; the R Foundation, St. Louis, MO, USA) was used to calculate PCC to analyze the correlation between DRGs and DRLs. The chi-squared test was used to examine differences in clinical features. Univariate and multivariate Cox regression analysis and LASSO regression were performed to construct the optimal DRLPS for HCC. Univariate and multivariate Cox regression analysis was used to calculate Hazard Ratio (HR) with 95% confidence interval. CIBERSORT and ssGSEA algorithms was used to assess immune infiltration and function. P value < 0.05 was reckoned as statistically significant.

### Supplementary Information

Supplementary Figures.

Supplementary Tables.

## Data Availability

The original contributions presented in the study are included in the article/ Supplementary Material. Further inquiries can be directed to the corresponding authors.
