# Supplementary material for: Machine learning-based disulfidptosis-related lncRNA signature predicts prognosis, immune infiltration and drug sensitivity in hepatocellular carcinoma
Source: Sci Rep. 2024 Feb 22;14:4354. doi: 10.1038/s41598-024-54115-8 (PMC10883983; doi:10.1038/s41598-024-54115-8)
Supplement: Supplementary file 1 — Supplementary Figures. [file 41598_2024_54115_MOESM1_ESM.docx]

**Supplementary information**

**Machine Learning-Based** **Disulfidptosis-Related lncRNA** **Signature Predicts Prognosis,** **Immune Infiltration and Drug Sensitivity in** **[Hepatocellular Carcinoma](javascript:;)**

Lei Pu, Yan Sun, Cheng Pu, Xiaoyan Zhang, Dong Wang, Xingning Liu, Pin Guo, Bing Wang, Liang Xue, Peng Sun


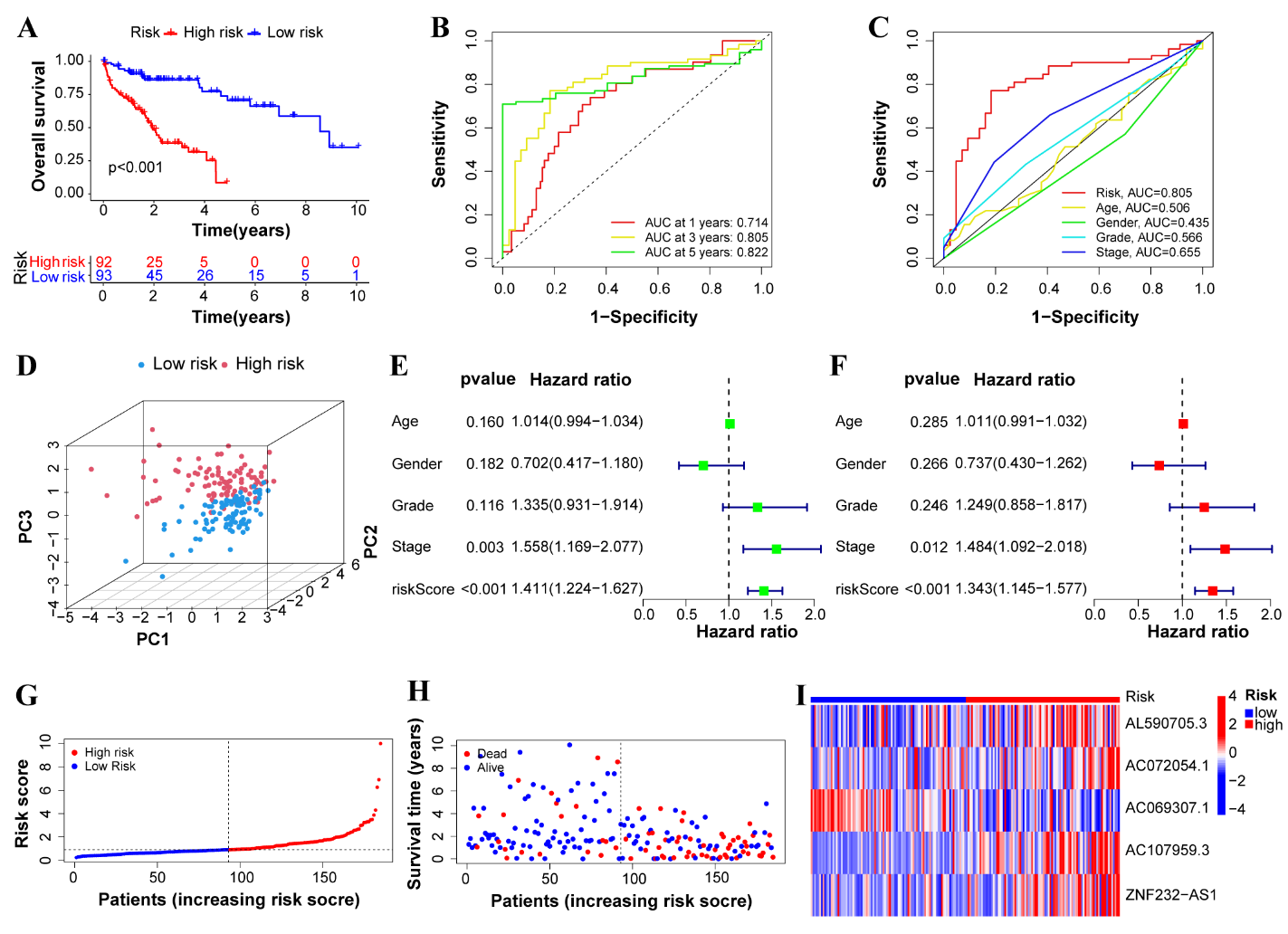


**Supplementary Fig S1**. Prognostic value of DRLPS in HCC patients. **(A)** K–M analysis of OS in the training set. **(B)** Time-ROC analysis of the DRLPS in predicting 1-, 2-, and 3-year OS in the training set. **(C)** AUC of ROC curves comparing the prognostic accuracy of the risk score and other clinical features. **(D)** Principal component analysis plot of the training set. **(E)** Univariate Cox regression analysis of clinical features and risk score. (**F)** Multivariate Cox regression analysis of clinical features and risk score. (**G-I)** The risk score plot, scatter plot of OS status and heat map of DRLs in the training set in the high- and low-risk groups.


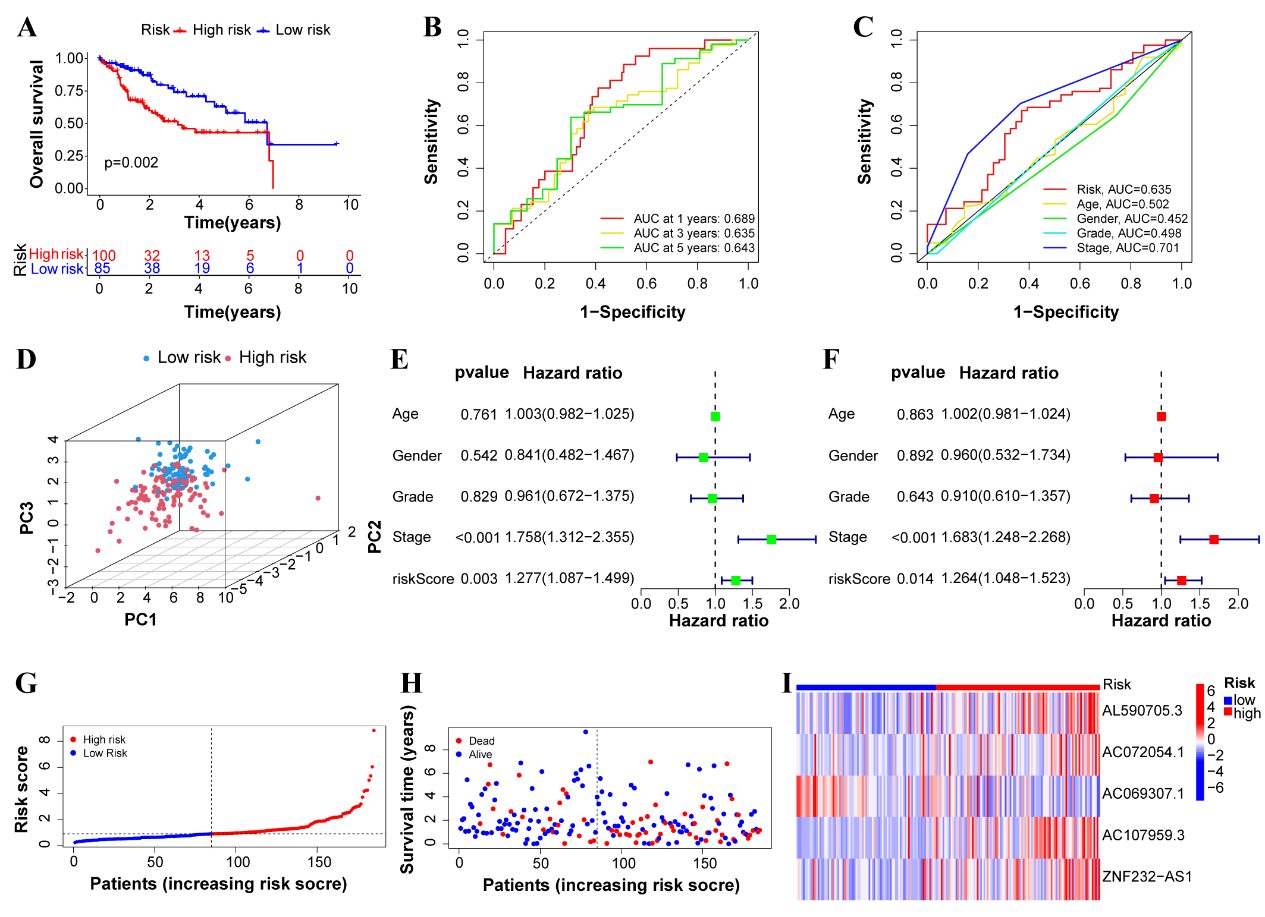


**Supplementary Fig S2**. Validation of DRLPS-based risk model based on the testing set. **(A)** K–M analysis of OS in the testing set. **(B)** Time-ROC analysis of the DRLPS in predicting 1-, 2-, and 3-year OS in the testing set. **(C)** AUC of ROC curves comparing the prognostic accuracy of the risk score and other clinical features. **(D)** Principal component analysis plot of the testing set. **(E)** Univariate Cox regression analysis of clinical features and risk score. **(F)** Multivariate Cox regression analysis of clinical features and risk score. **(G-I)** The risk score plot, scatter plot of OS status and heat map of DRLs in the testing set in the high- and low-risk groups.


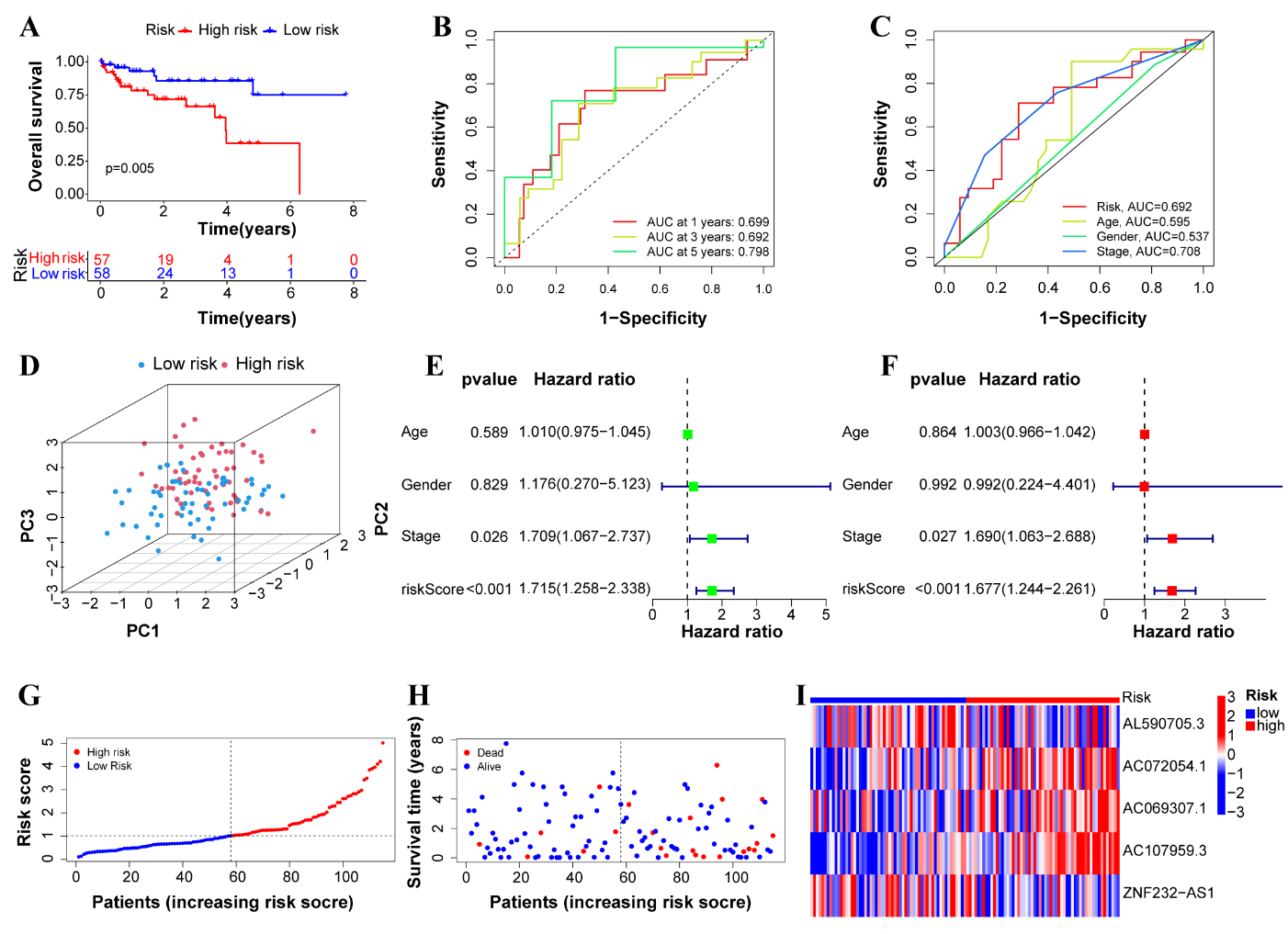


**Supplementary Fig S3**. The performance of DRLPS with GSE76427 as an external dataset in GEO database. **(A)** K–M analysis of OS in the GSE76427 cohorts. **(B)** Time-ROC analysis of the DRLPS in predicting 1-, 2-, and 3-year OS in the TCGA-LIHC entire cohorts. **(C)** AUC of ROC curves comparing the prognostic accuracy of the risk score and other clinical features. **(D)** Principal component analysis plot of the GSE76427 cohorts. **(E)** Univariate Cox regression analysis of clinical features and risk score. **(F)** Multivariate Cox regression analysis of clinical features and risk score. **(G-I)** The risk score plot, scatter plot of OS status and heat map of DRLs in the testing set in the high- and low-risk groups.
